# Discrete Particle Swarm Optimization Routing Protocol for Wireless Sensor Networks with Multiple Mobile Sinks

**DOI:** 10.3390/s16071081

**Published:** 2016-07-14

**Authors:** Jin Yang, Fagui Liu, Jianneng Cao, Liangming Wang

**Affiliations:** 1 School of Computer Science and Engineering, South China University of Technology, Guangzhou 510006, China; goodskyfly@163.com (J.Y.); lmwang@scut.edu.cn (L.W.); 2 School of Medical Information and Engineering, Guangdong Pharmaceutical University, Guangzhou 510006, China; 3 Data Analytics Department, Institute for Infocomm Research, 1 Fusionopolis Way, #21-01 Connexis (South Tower), Singapore 138632, Singapore; caojn@i2r.a-star.edu.sg

**Keywords:** discrete particle swarm optimization, wireless sensor network with mobile sinks, routing, energy efficiency

## Abstract

Mobile sinks can achieve load-balancing and energy-consumption balancing across the wireless sensor networks (WSNs). However, the frequent change of the paths between source nodes and the sinks caused by sink mobility introduces significant overhead in terms of energy and packet delays. To enhance network performance of WSNs with mobile sinks (MWSNs), we present an efficient routing strategy, which is formulated as an optimization problem and employs the particle swarm optimization algorithm (PSO) to build the optimal routing paths. However, the conventional PSO is insufficient to solve discrete routing optimization problems. Therefore, a novel greedy discrete particle swarm optimization with memory (GMDPSO) is put forward to address this problem. In the GMDPSO, particle’s position and velocity of traditional PSO are redefined under discrete MWSNs scenario. Particle updating rule is also reconsidered based on the subnetwork topology of MWSNs. Besides, by improving the greedy forwarding routing, a greedy search strategy is designed to drive particles to find a better position quickly. Furthermore, searching history is memorized to accelerate convergence. Simulation results demonstrate that our new protocol significantly improves the robustness and adapts to rapid topological changes with multiple mobile sinks, while efficiently reducing the communication overhead and the energy consumption.

## 1. Introduction

Recently, wireless sensor networks (WSNs) have gained enormous attention for their wide range of applications [1]. WSNs consist of many tiny sensor nodes and one or multiple sinks, sensor nodes gather data from the sensing environment to sinks by communicating with each other. Energy efficiency is the most important issue in WSNs due to the limited battery capacity of sensor nodes. In WSNs with static sinks, the nodes close to the sinks would become hotspots and die earlier than others, because they are more likely to be the intersection of multihop routes and need to deplete their battery supplies to transmit huge amounts of data for other sensor nodes to the sinks [2]. Node death will result in a series of problems, such as disruptions in the topology, reduction of sensing coverage and packets loss, and so on. Therefore, routing protocols designed for WSNs have to incorporate load-balancing in order to achieve uniformity of energy consumption throughout the network. Mobile sinks are a good solution to avoid the hotspots [3,4]. In mobile based routing protocols, the hotspots around the sink change as the sink moves, which means that each node in the network has the chance to become the neighbor of the sink, so the high energy drainage around the sink is spread through the network, which helps achieving uniform energy consumption, and the network lifetime can be prolonged.

However, due to the sink mobility, the paths between source nodes and sinks are frequently updated, which introduces significant energy overhead. Therefore, mobile sink routing protocols should minimize the energy overhead of such operations while avoiding an extremely high increase of the sensor data delivery latency. A lot of distributed mobile sink protocols are proposed. Among these, the hierarchical protocols [2,5,6] are the most important and the most widely adopted, which aim to decrease the load of advertising the sink’s position to the network by constructing a virtual multitier (two or more) hierarchy role among the nodes. The high-tier nodes that can be easily reachable obtain and store the fresh sink position, while the remaining nodes query them to acquire the sink position whenever needed. Such a scheme does not need the network-wide sink advertisement, and thus significantly decreases the communication overhead and enhances the energy-efficiency of the network. However, the high-tier nodes may become hotspots. Yet the non-hierarchical protocols utilize mechanisms like flooding, overhearing or exploit geometric properties to advertise the sink’s position to the source nodes [7,8]. Thus, they can eliminate the overhead of constructing the virtual hierarchical structure and the possibility of hotspots on such a structure. However, they need the network-wide sink advertisement. The review of prominent hierarchical and non-hierarchical mobile sink routing protocols are presented in [9].

Recently, centralized nature-inspired algorithms have been studied to design routing protocol for MWSNs and have been proven to be good a solutions [10,11,12,13]. PSO is now prevailing due to its simple concept, easy implementation and effectiveness [14]; therefore, in this study, we employ PSO to build the optimal routing tree. In PSO-based protocols, routing establishment is formulated as an optimization problem; mobile sink masters the topology of the entire network by using network-wide flooding and runs various improved PSO to build the optimal routing paths for all source nodes. However, there are some shortages in existing schemes: (1) PSO is originally designed for continuous optimization problem, thus, they cannot deal with the discrete optimization problem as well as continuous optimization problems. Yet the routing optimization problem in MWSNs is discrete, and none of the existing solutions attempt to solve this problem. Therefore, the main goal of this paper is to propose a new discrete PSO algorithm by adapting the conventional PSO to build the optimal path tree more effectively in MWSNs; (2) Network-wide flooding significantly increased the communication overhead and energy consumption, while choosing the optimal routing result from all nodes greatly increases the computation burden. Thus, the second goal of this paper is to design a more efficient routing strategy; (3) As an evaluation criterion which helps us to periodically update the particles’ personal best position *Pbest* and the swarm’s global best position *Gbest*, fitness function is very important, because it directly affects the final results. Good fitness function improves the solution. In order to achieve better network performance, the existing fitness functions focus on maximizing the total remaining energy, and minimizing the total route length, total energy consumption and total communication delay of the candidate routing result. No attempt has considered such a case that the lifetime of a routing path just only depends on the relay node that has least lifetime, rather than the total remaining energy and total energy consumption. Therefore, the third goal of this paper is to give a better fitness function for our new routing protocol.

In this paper, we present an efficient routing strategy in which the greedy forwarding mechanism is employed as the underlying routing solution and the unicast local flooding method, rather than network-wide flooding, is adopted to reduce the communication and energy overhead caused by frequent broadcast communications. In our strategy, building routing tree from some source nodes to a mobile sink is formulated as an optimization problem, and the novel GMDPSO algorithm is put forward to solve this optimal problem. The main highlights of the proposed GMDPSO are as follows. First, we give a new fitness function in which we carefully calculated the energy consumption of relay nodes and try to minimize the distance among them to achieve energy conservation. Besides, we also minimize the total communication delay of routing tree. Second, the particle’s velocity and position of conventional PSO have been carefully redefined under discrete scenario so as to make them as easy as possible to encode/decode. Third, to effectively reduce the exhaustive global searching space and drive the particles to promising regions, the particle’s velocity and position updating principles have been thoroughly reconsidered by taking the advantage of the network topology of the collected candidate relay nodes by sink. Fourth, to avoid being trapped into local optima when building each optimal routing path, a greedy searching strategy specially designed for the particles to adjust their positions is proposed by improving the greedy forwarding mechanism. Fifth, searching history is memorized to accelerate convergence. By employing the provided GMDPSO and new fitness function, our new routing protocol is able to find the optimal routing tree, which well balances energy consumption of the relay nodes, delay and the overall rout tree length.

In summary, the contribution of this paper can be listed as follows.

- An efficient routing strategy of the MWSNs is designed which is formulated as optimization problem.
- A novel greedy discrete PSO with memory (GMDPSO) algorithm, which offers faster global convergence and higher solution quality, is put forward to quickly build the optimal routing tree, which can decrease the control overhead and minimize energy consumption.
- Our new routing protocol is realized based on GMDPSO and more accurate new fitness function.
- Simulations of the proposed protocol are performed to demonstrate its performance against some of the existing protocols.

The rest of the paper is organized as follows. The related work is presented in Section 2. Section 3 states the system model, which includes network model, energy model and fault model, and used terminologies. In Section 4, we present the routing strategy and the flowchart of our protocol. Section 5 gives a detailed description of the proposed GMDPSO, which is the core algorithm of our routing protocol. Section 6 evaluates the performance of our protocol by comparing it with other routing protocols. Finally, Section 7 concludes the paper.

## 2. Related Work

Various routing protocols have been proposed for MWSNs. TTDD (a Two-Tier Data Dissemination) [15] initially builds a rectangular grid structure which divides the network into cells with several dissemination nodes that are used to relay the query and data to/from the proper source. Whenever sinks request data, they query the network by local flooding within the cell and the query packets are relayed to the source nodes through dissemination nodes. A data path from the source to the sink is then established using the reverse of the path taken by the data request. However, TTDD suffers from the high overhead of constructing a separate grid for each source node. Unlike TTDD, Grid-Based Energy-Efficient Routing From Multiple Sources to Multiple Mobile Sinks (GBEER) [16] constructs a single combined rectangular grid structure for all possible sources to eliminate the high overhead of constructing separate grids for each source. However, the frequent grid change due to solving the hotspots introduces extra traffic on numerous nodes residing between the crossing points. In place of a rectangular grid, HPDD (Hexagonal Path Data Dissemination) [17] utilizes a common grid structure composed of hexagons. Which can provide shorter data and sink advertisement routes, but it suffers from the same hotspot problem. SEAD (Scalable Energy-efficient Asynchronous Dissemination) [18] constructs a minimum-cost weighted Steiner tree for the mobile sink by considering the distance and the packet traffic rate among nodes to save communication energy. Like TTDD, separate trees are constructed for each source; thus, the overhead of establishing such trees is very high. Ring Routing [19] designs an easily accessible ring structure to mitigate the anticipated hotspot problem with low overhead and minimize the data reporting delays. However, its questionable scalability is not good. Besides, the overhead of the initial ring construction for large or sparse networks will be high.

In agent-based schemes, one or more agents, which take on the role of representatives for the sources or the sink, are selected to relay the traffic between sources and the sink. These protocols usually utilize infrequent flooding to advertise the location of the agents. IAR (Intelligent Agent-Based Routing Protocol) [20] provides efficient data delivery to mobile sinks in large scale wireless sensor networks with sink mobility. IAR reduces the packet loss and signal overhead, meanwhile improves degraded route called triangular routing problem. However, as the hotspot, the agent (or IR) is chosen only depending on the distance to the sink without considering its remaining energy. Thus, once a node with low remaining energy is chosen to be the agent, it may die quickly, which leads to break the existing path to the sink. DHA (Data Dissemination Protocol Based on Home Agent and Access Node) [21] employs Home Agent and Access Node to design a simple protocol. Similar to IAR, the load on the home agent is immense, and changing the home agent requires global flooding. To decrease the frequency of global floods, SinkTrail [7] constructs and utilizes a logical coordinate space, which does not require position aware sensors and enables simple data dissemination by logical greedy geographic routing. However, the sink’s mobility is assumed to be nomadic and limits the applicability of the protocol. VGE-MGP [22] is another efficient mobile sink based routing without the physical geographic information.

Recently, nature-inspired algorithms are applied to solve the problem of the mobile based protocol. SIMPLE (swarm intelligence based protocol) [23] is based on a swarm agent that integrates the remaining energy of nodes into the route selection mechanism and maximizes the network’s lifetime by evenly balancing the remaining energy across nodes and minimizing the protocol overhead. The protocol is robust and scales well with both the network size and in the presence of multiple sinks. The movement of sinks may lead to the breakage of the existing routes. In most routing protocols, the query packets are broadcasted to repair a broken path from source node to sink, which causes significant communication overhead in terms of both energy and delay. In order to repair the broken path and maintain the available route from the source nodes to the mobile sink through a multi-hop network with lower communication overhead, IOLPSOR (immune orthogonal learning particle swarm optimization algorithm based routing recovery method) [11] is proposed to provide more efficient routing recovery capability to MWSNs. Furthermore, a more efficient ECPSOAR (efficient routing recovery protocol with endocrine cooperative particle swarm optimization algorithm) [10] is proposed to repair the broken path caused by both the movement of the sink and failure of sensor nodes. However, neither IOLPSOR nor ECPSOAR considers the following two cases. One case is that, the lifetime of the routing tree depends on the relay node that has the least lifetime. The other case is that, the improved PSOAs used by these two protocols do not well fit the discrete routing scenario.

## 3. System Mode and Terminologies

### 3.1. System Mode

We assume such a MWSN scenario: A wide open area is covered with a large number of homogeneous sensor nodes; there are *N* source nodes and *M* mobile sinks. Sinks can move anywhere within the sensor field, at any time with a fixed speed. Each source node sends its sensed data through multi-hops to the nearest sink via one and only one routing path. Several nodes can route packets to the mobile sink. In our network mode, individual node only owns the local information, such as its unique ID, residual energy level, delay and the distances to its neighbors, which can be estimated based on the received signal strength [24,25]. Our model does not need any extra positioning system such as GSP.

We use the same energy model and fault model proposed in [10]. The energy consumption equation of sensing and transferring m bit data is as follows:

*E_sens_*(*m*) = *α_1_* × *m* (1)

*E_tx_*(*m*, *d*) = (*β_1_* + *β_2_* × *dis*(*i*, *j*)*^n^*) × *m* (2)

*E_rx_*(*m*) = *γ_1_* × *m* (3)

where *dis* (*i*, *j*) is the distance between node i and node j, *E_sens_* is the energy consumption of sensing m bits of data, *E_tx_*(*m*, *d*) and *E_rx_*(*m*) are the energy consumption of sending and receiving *m* bits of data, *n* is the channel attenuation index, *α_1_*, *β_1_*, *β_2_* and *γ_1_* are energy consumption parameters of sensing circuit, sending circuit, sending amplifier and receiving circuit, respectively. Let *P_fault_* be the probability of sensor node’s failure in the network. We assume that the probability of the mobile sink failure is approximately 0. If any relay node is failed, it is handled by our routing algorithm.

### 3.2. Terminologies

Throughout this paper, we use the following terminologies:

- $Source_{j}$ the *jth* sensor node. $Sink_{i}$ is the *ith* mobile sink.
- $Agent_{i}$ is the agent node belonging to $Sink_{i}$. One mobile sink has just only on agent node
- *N* is the number of source nodes and *M* is the number of mobile sinks
- R is the communication range of sensor node.
- $RN_{j}^{k}$ is the *kth* relay node on the path *Path_j_*. $RN_{j}^{k+1}$ is the next relay node of $RN_{j}^{k}$ and$\;RN_{j}^{k-1}$ is the previous relay node of $RN_{j}^{k}$.
- $N_{Path_{j}\;}$ is the number of relay nodes in $Path_{j}$. $N_{Path}^{Sink_{i}}$ is the number of routing paths of$\;Sink_{i}$.
- $Path_{j}$ is the path from *jth* source node in the network to its nearest mobile sink $Sink_{i}$, $Path_{j}$ = {*Source_j_*, $RN_{j}^{1},RN_{j}^{2},\ldots ,RN_{j}^{k}\ldots ,RN_{j}^{N_{Path_{j}}},Agent_{i},Sink_{i}$}.
- $PTree_{i}$ is the routing tree of $Sink_{i}$.$\;Path_{j}\in PTree_{i}$ and $RN_{j}^{k}\in PTree_{i}$.
- $lf(RN_{j}^{k})$ means the lifetime of the $RN_{j}^{k}$. $lf(PTree_{i})\;$means the lifetime of the $lf(PTree_{i})$.
- $Delay(RN_{j}^{k}$) is the delay of the $RN_{j}^{k}$. $Delay\left(PTree_{i}\right)$ means the overall delay of the $PTree_{i}$.
- *Len*($Path_{j})$ means the length of the $Path_{j}$. *Len*($PTree_{i})$ means the overall length of the $PTree_{i}$.
- $E_{cons}(RN_{j}^{k})$ is the energy consumed by $RN_{j}^{k}$ due to data forwarding.
- $E_{cons}^{Path}\left(Path_{j}\right)$ is the total energy consumption of routing path $Path_{j}$.
- *G*(*V*,*E*) or *G* means the connected graph containing all candidate relay nodes.
- *Set*(*V*) is the set of all sensor nodes of *G*(*V*,*E*), i.e., the set of all candidate relay nodes.
- *N_V_* is the number of sensor nodes in *Set*(*V*). $N_{V}^{\prime }\;$is the number of sensor nodes in *Set*(*V*) except the agent node, $\;N_{V}^{\prime }=N-1$, i.e., the number of *Set*(*V*)-{$Agent_{i}$}.
- *Neig*(*node_i_*) is the set of all those sensor nodes within the communication range of *node_i_*, therefore, *Neig*(*node_i_*) = {*node_j_*|0 < *dis*(*node_i_*, *node_j_*) ≤ R}.
- *NextRelay*(*node_d_*) is the sensor node which is selected as the next relay node for *node_d_*. Therefore, *NextRelay* (*node_d_*) = {*node_j_*|∀*node_j_ dis*(*node_j_*, *Agent*) < *dis*(*node_d_*, *Agent*)}.

## 4. New Routing Protocol for MWSNs

In this section, we design an efficient routing strategy, based on the routing strategy, a new routing protocol is presented.

### 4.1. Our Efficient Routing Strategy in MWSNs

In our routing strategy, mobile sinks employ agent-based data gathering scheme [20,26,27] to collect sensed data. Our routing strategy can be described as follows.

#### 4.1.1. Design of the Neighbor Table

Above of all, we design the neighbor table that is the key component of our protocol. It is illustrated in Table 1.

self_ID means a node itself, neighbor_ID means its neighbor node, Dis is the distance between seft_ID and neighbor_ID. isRelay is a Boolean flag indicating whether the node pair <self_ID→neighbor_ID > within a routing path, isRelay = 1 means true and data packet is routed from self_ID to neighbor_ID, its initial value is set to 0. Table 1 is the neighbor table for node 7 and illustrates that node 7 has two neighbor nodes: node 3 and node 9, the distance between node 7 and node 3 is 20, the distance between node 7 and node 9 is 15, in addition, <7→3> within a routing path.

Notably, our neighbor table is a wonderful issue. Some advantages can be summarized as follows: Firstly, neighbor table is the basic component of our protocol. Its basic function is used to record neighbor information which is the key information used to build the optimal routing path. Secondly, neighbor table enhances the MWSN scalability and the protocol robustness. When some nodes are failed, they will be deleted from its neighbors’ neighbor table on demand, and never be used to build the new optimal routing path. If the failed nodes are repaired or replaced, or new nodes are added in the MWSNs, corresponding neighbor information will be inserted into their neighbors’ neighbor table, thus, they can be used to build the new optimal routing path. Beyond that, when the routing path is changed, it is only to reset the isRelay flag of the relevant nodes, and this feature is well suited to resolve the routing problem in MWSNs. Thirdly, the isRelay flag indicates the routing information. It serves two purposes: routing tree establishment and locating the failed nodes. The routing tree is established by resetting the isRelay flag. In addition, if a relay node has not received any packet from its previous relay node after a reasonable time interval, it implies that the previous relay node has failed. With the help of the isRelay flag, the relay node can quickly find failed node ID from its neighbor table.

#### 4.1.2. Building Routing Tree

Here, we detailed the procedure of building the routing tree from source nodes to sink. This procedure consists of the following steps:

***Step 1*** *Network initiation and neighbor discovery.* After all sensor nodes are deployed in the sensed field, all nodes can update their neighbor table using RSSI-based distant estimated scheme.

**Figure 1:**
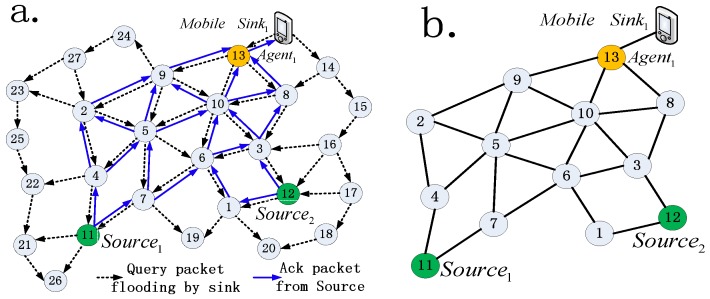
Illustration of (**a**) sink queries MWSN; (**b**) graphic description of MWSN.

***Step 2*** *Collect network statuses*. When a sink move in a target area, the first thing has to be completed is to find the appropriate agent by local-broadcasting 1-hop Agent Query packet (AQ). The sink selected the closest node as the agent, and then broadcasts a Data Query (DQ) packet using unicast local flooding. Each node that receives the DQ packet firstly forwards the DQ to its neighbors and then updates its neighbor table if necessary. When a source matches DQ packet, it replies a Success Response (SR) packet to the sink. Each node that receives the SR packet firstly forwards the SR to its neighbors, then broadcast Data Query Response (DQR) packet that includes its newest state information such as residual energy, delay, and neighbor table data and so on along with its ID to the sink. Once the SR packet is received by sink, the data query procedure is finished immediately or finished after a reasonable time from then. This procedure as illustrates as Figure 1a. There are two special operations in this process are noteworthy. One is that all query packets (i.e., AQ and DQ) and their reply packets (i.e., DQR) are unicast by local flooding to reduce the overall communication and energy overhead. The other one is that, when a source node receivs multi DQ packets from multi sinks, it only replies to the first received DQ packet, which can assure it only sends its sense data to the nearest mobile sink to save energy.

**Figure 2:**
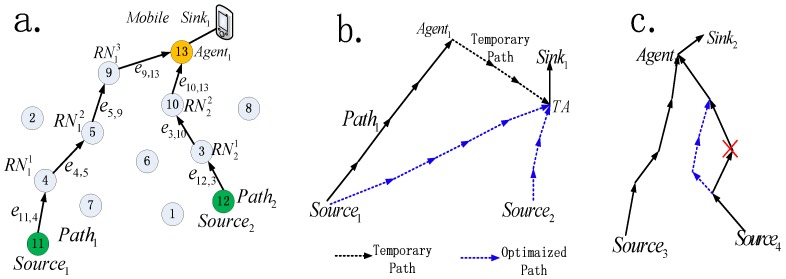
(**a**) Illustration for routing tree; (**b**) Routing recover for mobile moves away; (**c**) Routing recover for relay node failed.

***Step 3*** *Build optimal routing tree*. In most situations, multi-source nodes send data packets to a same sink, therefore, we need to build a routing tree. Root node is the agent, and the leaf nodes mean its source nodes. An example of routing tree is shown in Figure 2a. There are two routing paths, i.e., {*Path_1_*, *Path_2_*}, in the routing tree, the root node is *Agent_i_* and the leaf nodes are *Source_1_* and *Source_2_*. The routing tree contains 5 relay nodes. Once finished ***Step 2***. Sink has collected a number of neighbor tables along with their remaining energy information and delay information which are wrapped in DQR packets. As illustrated in Figure 1b, the network topology of candidate relay nodes that extracted from the neighbor tables, i.e., *G*(*V*, *E*), can be constructed by sink. Where *e_i_*∈*E* indicates that two adjacent sensor nodes can communicate with each other. *G*(*V*, *E*) is stored as adjacency matrix *A*, *A*[*i*, *j*] can be calculated as follows:

(4) $$A\left[i,j\right]=\left\{ \begin{array}{l} 0\;if\;i=j\;\\ dis\left(i,j\right)\;dis\left(i,j\right)\leq R\;\\ \infty \;if\;dis\left(i,j\right)>R\; \end{array}\right.$$

where *i*, *j* are two adjacent nodes, and *dis*(*i*, *j*) denote the length of *e_ij_*∈*E*.

Each optimal routing path from each source node towards the sink can be built by using the improved greedy forwarding mechanism from candidates based on the network topology. Notably, even though each routing path (or branch) of a sink is optimal, the routing tree of the sink may not be the optimal due to the intersection relay nodes shared by multi paths. Therefore, the optimal algorithm needs to be employed to achieve the global optimum (the optimal routing tree). We designed GMDPSO , which is detailed in Section 5, to solve the problem.

***Step 4*** *Establish routing tree and begin data transmission*. After a mobile sink finished building the optimal routing tree in ***Step 3***, it sends Routing Result (RR) packet, in which the routing tree can be stored as array, singly linked list or other forms, to its source nodes to establish the routing tree. The routing path establishment process can be detailed as follows: Assuming that *Path_j_* = {*Source_j_*→11→9→7→3→$Agent_{i}$→$Sink_{i}$} is a routing path of the routing tree.$\;\;Sink_{i}$ sends RR packet to its agent. When $Agent_{i}$ received the RR packet, it firstly search its previous relay node from the received packet according its ID, i.e., node 3. Then it sets isRelay = 1 for node pair {$Agent_{i}$→node3} in its neighbor table. Next, $Agent_{i}$ replaces the destination address of RR packet with node3 and forwards it to node3. After received the RR packet, node 3 forwards it to its previous relay node7 as the same way, and so on for each relay node in the routing path. Finally, the source *Source_j_* received the RR packet, which means that the routing path is established completely and source can begin the data packet transmitting via the reverse path of RS packet towards source node. The detailed data forward process is described as follows: *Source_j_* generates a data packet and queries the neighbor_ID from its neighbor table as its next relay node using the condition isRelay = 1, i.e., node11, then the data packet is send to node11. After received the data packet, node 11 immediately queries its next relay node (i.e., node 9) and forwards data packet to node 9 in the same way, and so on for each relay node in the routing path. Finally, all data packets from *Source_j_* are received by $Agent_{i}$, and then $Sink_{i}$ receives data packet from $Agent_{i}$ directly.

#### 4.1.3. Routing Path Recovery

When sink moves out of the radio rang of agent or relay node fails, the original routing path need to be recovered to continue the data gathering. The routing recover procedure is detailed in two cases:

(1) When sink moves out of the radio rang of agent.

$Sink_{i}$ cannot receive any packet from $\;Agent_{i}$, then it needs to select a new agent and build a new routing path to continue the data gathering. To ensure no previous data packet is lost, Temporary Agent node (TA) is introduced in our routing strategy. This routing recovery result is shown in Figure 2b. In order to help the sink to judge whether or not it is within the radio rang of an agent, the agent and relay nodes are required to send at least one packet to sink for a time interval *T*. Therefore, even though they have nothing to send, they also need to send a NULL packet to sink. Therefore, if not any packet has been received from $Agent_{i}$ for time interval *T*, $Sink_{i}$ can knows that it moves away from $\;Agent_{i}$. To continue the data gathering, the following steps need to be completed:

***Step 1*** Select the TA.

Sink selects the closest node as the TA. If the sink moves faster, then TA is farther away from $\;Agent_{i}$ ,which means that more relay nodes are required between TA and $\;Agent_{i}$.

***Step 2*** Build the temporary routing path

After TA is selected, $\;Sink_{i}$ sends Temporary Routing Path Setup (TRPS) packet to $Agent_{i}$ via TA. Once received the TRPS packet,$\;Agent_{i}$ sends its cached data packets which come from source *Source_j_* to TA along the reverse path of TRPS packet, the reverse path is named Temporary Path (TP). Because some data packets will be lost after the sink moved away from $Agent_{i}$ and before TP is established, the $Agent_{i}$ needs to cache the data packets in this time interval to avoid data loss. Once the $Agent_{i}$ received the TRPS packet, these cached data packets are routed to TA via TP.

***Step 3*** Collect network statuses.

The procedure is the same to ***Step 2*** in Section 4.1.2. The only difference is that the sink sends Routing Path Reset (RPRS) packet to source *Source_j_* to collect the newly network status information.

***Step 4*** Build optimal routing path.

As same as ***Step 3*** in Section 4.1.2. The only difference is that the optimal routing tree is built for the nearest source nodes using those nodes whose response packet (RPRSR) are received by sink.

***Step 5*** Establish routing tree and begin data transmission.

The procedure is the same as ***Step 4*** in Section 4.1.2.

***Step 6*** Clear original routing path.

Notably, when the new optimal routing path established, the original routing path is still work on. Before source node begin routing data packet via new optimal routing path, it sends Routing Path Clear (RPC) packet to TA along the original routing path and TP, each relay node on the path that received RPC packet will reset its isRelay = 0 to remove routing state information. Once the original routing path and TP is cleared, TA becomes the new agent $Agent_{i}$.

(2) When the relay node fails

Now we consider the relay node fault tolerant. This routing recovery procedure is shown in Figure 2c.

***Step 1*** Locate the failed node

When a relay node (e.g.,$\;RN_{j}^{k}$) in *Path_j_* = {*Source_j_*,$\;RN_{j}^{1},\;\;RN_{j}^{2},\;\ldots ,\;RN_{j}^{k}\ldots ,\;RN_{j}^{n},\;Agent_{i},\;Sink_{i}$} has failed, e.g., exhausted its battery power, then *Path_j_* is broken, its next relay node $RN_{j}^{k+1}$ can realize this situation if it cannot receive any packet from $RN_{j}^{k}$ for a predetermined time interval *T*. Once detecting routing path is broken, $RN_{j}^{k+1}$ immediately queries its neighbor table to obtain the failure node ID (i.e., $RN_{j}^{k}$) and sends a relay node failure (RNF) packet, which includes the failure node ID to $Agent_{i}$ via the original path. The relay node in the original path that received the RNF packet, e.g.,$\;RN_{j}^{k+2}$, forward it to the next relay node ($RN_{j}^{k+3}$) and reset its isRelay = 0 to remove routing information.

***Step 2*** Build new routing path without failed node

After received the RNF packet, $Agent_{i}$ removes the failure node $RN_{j}^{k}$ from the set of all candidates *Set*(*V*) and executes our new protocol to re-build new optimal routing path with *Set*(*V*)-{$RN_{j}^{k}$}. The subsequent steps are the same as routing recover for mobile moves away the current agent, which are described in Section 4.1.2.

### 4.2. Overview of New Protocol

Based on our efficient routing strategy, a new centralized discrete PSO routing protocol is presented to solve the problem of routing in MWSNs.

**Figure 3:**
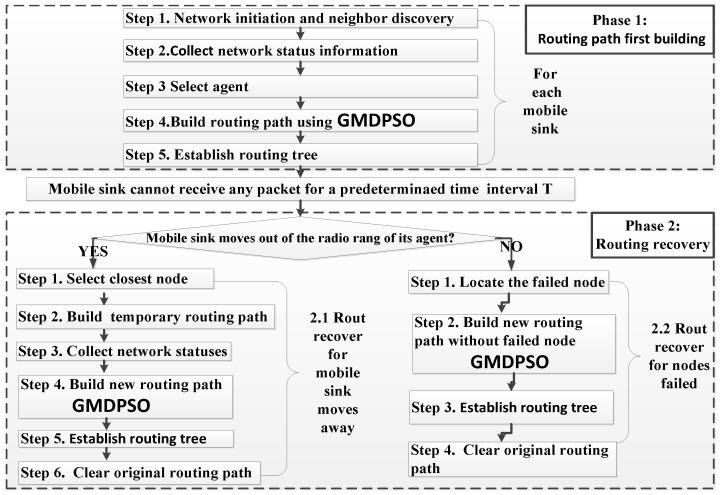
Flowchart of new routing protocol.

The new protocol consists of two phases, the various steps are depicted in the flowchart as shown in Figure 3. When a mobile sink moves in the sense area, the routing tree from nearest source nodes towards it will be established in phase 1. When a mobile sink moves away its agent, or its relay node fails, its original routing path is recovered in phase 2. The detailed description can be seen in Section 4.1.

It can be seen from Figure 3 that the GMDSPO is the core algorithm of our new protocol, which is used to build the optimal tree from the nearest source nodes to the mobile sink in both phase 1 and phase 2.

## 5. The Proposed GMDPSO for Our Protocol

### 5.1. PSO

PSO is a population-based stochastic searching algorithm, which is inspired by social behavior of bird flocking and fish schooling. The easy implementation, concise framework and fast computational convergence make PSO a popular optimization technique for solving continuous optimization problems.

PSO works with a swarm of a predefined size (say $N_{p}$) of particles. Each particle has a position and a velocity vector. The position vector gives a complete candidate solution to the optimization problem, and the velocity vector denotes the position-changing tendency. Each particle is evaluated by a fitness function to judge the quality of the solution to the problem .To search for the optimal solution, a particle iteratively updates its flying velocity and current position according to its own flying experience, i.e., personal best called *Pbest_i_* and according to the other particles’ flying experiences, i.e., global best called *Gbest*.

In canonical PSO, a particle updates its position and velocity using the following simple rules:

(5) $$V_{i}\left(t+1\right)=w\times V_{i}\left(t\right)+c_{1}\times r_{1}\times \left(Pbest_{i}-X_{i}\left(t\right)\right)+c_{2}\times r_{2}\times \left(Gbest-X_{i}\left(t\right)\right)$$

(6) $$X_{i}\left(t+1\right)=X_{i}\left(t\right)+V_{i}\left(t\right)$$

where *V_i_* = {$v_{i}^{1}$,$v_{i}^{2}$, …, $v_{i}^{D}$} and *X_i_* = {$x_{i}^{1}$, $x_{i}^{2}$, $1_{I}^{3}$, …, $x_{i}^{D}$} are the ith particle’s (say $P_{i}$) velocity and position vector. *w* is the inertial weight. *t* means the current iteration and *t +* 1 is the next itreration. *c_1_* and *c_2_* are acceleration factors term as cognitive and socail componeents. *r*_1_ and r_2_ are two different uniformly distributed random numbers in the range [0,1]. *Pbest_i_* = {$pbest_{i}^{1}$, $pbest_{i}^{2}$, $pbest_{I}^{3}$, …, $pbest_{i}^{D}$} is the personal best position of $P_{i}$ and *Gbest* = {*gbest^1^, gbest^1^, …, gbest^D^*} is the global best position of the whole swarm.

Conventional PSO was designed to optimize continuous problems. However, its fast convergence and easy implementation yet effectiveness have driven us to extend continuous PSO to solve the discrete routing optimization problem in MWSNs.

### 5.2. GMDPSO: Greedy Discrete PSO with Memory

The GMDPSO makes use of the network topology of candidate relay nodes to guide particle’s position and velocity updates. The greedy strategy originated from greedy forwarding mechanism is proposed to direct particles to move towards better position. Some small operators, such as position initialization and memory mechanism, are introduced to speed up convergence. This section will describe the proposed algorithm in detail. The whole framework of GMDPSO is given in Algorithm 1.

**Algorithm 1** Framework of GMDPSO Algorithm**Parameters:** particle swarm size *N_p_*, number of iterations *Gen*, inertia weight *w*, learning factors *c_1_* and *c_2_*;**Input:**
(1) adjacency matrix *A* for *Sink_i_*; (2) the residual energy vector *E_res_* = {*E_res_* (1), *E_res_* (1), …, *E_res_* (*N −* 1)}(3) the delay vector *Del* = {*Del*(1), *Del*(2), …, *Del*(*N* − 1)};**Output:**
*Gbext.X*: the routing result**Step 1:** initialize***Prev*****Step 2: *for***
*i* = 1 to *N_p_*
***do***//I**nitialize the population***P*[*i*]*. X* = initPathTree // seeing Section 5.2.3 for more information***Prev*** = ***Prev***∪*P*[*i*]*.X **If***
*P*[*i*]*.X*∉***Prev***// memorize new position*P*[*i*]*. V* = 0*Pbest*[i] = *P*[*i*]Calculate Fitness P[i]. fit //Using Equation (26)***end*****Step 3:**
***G****best* = {*P*[*k*]|*Fitness(Pbest*[*k*]) = min(*Fitness(Pbest*[*i*]))}**//update *G****best***Step 4:**
***While (!(Terminate))******for***
*i* = 1 to *N_p_*
***do*****Update**
*P*[*i*]**//**carefully described in Section 5.2.4Memorize new position: ***Prev*** = ***Prev*** ∪ new *P*[*i*]*.X*
***If***
*P*[*i*]*. X*∉***Prev***Calculate Fitness P[i]. fit
Update the *Pbest_i_*
*Pbest*[*i*]*.X* = *P*[*i*]*.X*
**if**
*Pbest*[*i*]*.fit* > *P*[*i*]*. fit****end***Update the *Gbest*: *Gbest = Pbest*[*best*]***end*****Step 5:** output Gbest.X


It is worthy noted that the ***Prev*** variable is delicately designed in GMDPSO, it memorizes the search history to avoid the same position be updated more than once during the iterative process, and thus can avoid lots of reduplicative search and promote particle to find a better position faster. In other words, it can speed up the convergence of the algorithm. The function of ***Prev*** is called memory mechanism.

The positions are iteratively updated until the termination condition is satisfied. In our approach, there are two termination conditions: predefined iteration number and accuracy requirements. Once one of the two conditions is satisfied, the algorithm stops and the particle *Gbest* is found, *Gbest.X* represents the clustering result or routing result.

#### 5.2.1. Fitness Function Derivation

Fitness function is an important issue because it directly affects the final results. Now, we construct our fitness function (*fitness*) to evaluate the individual particle of population, in GMDPSO, the optimal tree is built such that it minimizes the *fitness value*. We have three objectives when build the optimal routing tree for *Sink_i_* (i.e., *PTree_i_*): **The first one** is to maximize the lifetime of the routing tree to achieve energy-consumption balancing; **The second one** is to minimize the length of the routing tree to achieve energy conservation and enhance reliability; and **the third one** is to minimize the communication delay to enhance network throughout. In order to tune the three objectives to build optimal routing tree, we need to normalize the above three objectives and use the weight sum approach (WSA) [28] to construct our multi-objective fitness function *fitness*. Therefore, our fitness function depends on the following parameters:

(1) Lifetime of relay node

Our first objective is to maximize the routing tree lifetime, which is defined as the time interval from the establishment of the routing tree till its first relay node dies (FND). This is possible if we can maximize the lifetime of relay node that has least lifetime. Let $RN_{j}^{k}$ be the kth relay node on the Pathj. The lifetime of $RN_{j}^{k}$ is defined as follows:

(7) $$lf\left(RN_{j}^{k}\right)=E_{res}\left(RN_{j}^{k}\right)/E_{cons}\left(RN_{j}^{k}\right)$$

where *Eres*($RN_{j}^{k}$) is the residual energy of relay node $RN_{j}^{k}$, and $E_{cons}(RN_{j}^{k})$ is its consumed energy. It can be seen from Equation (7) that, even though $RN_{j}^{k}$ keeps more *E_res_*($RN_{j}^{k}$), it maybe die faster if its $E_{cons}(RN_{j}^{k})$ is bigger than others at the same time.

Now, we begin the energy consumption analysis for relay node. $RN_{j}^{k}$ must consume energy to forward the incoming data packets, which come from source node whose routing path to sink goes through it. Before calculating the routing energy consumption, it is needed to calculate the total number of incoming packets, which come from other relay node toward sink. Since multi routing paths may share some relay nodes, the number of incoming packets can be recursively calculated as follows:

(8) $$N_{in}\left(RN_{j}^{k}\right)=\left\{ \begin{array}{l} 0\;if\;NextRelay\left(RN_{l}^{x}\right)\neq RN_{j}^{k},\;\forall l\;1\leq l\leq N_{path}^{sink_{i}},\forall RN_{l}^{x}\in Path_{l}\;\\ \sum \left\{N_{in}\left(RN_{l}^{x}\right)|NextRelay(RN_{l}^{x})=RN_{j}^{k}\right\},\;otherwise \end{array}\right.$$

The relay node $RN_{j}^{k}$ will consume its energy to receive *N_in_*($RN_{j}^{k}$) incoming packets and forward them. Therefore, the total data forwarding energy consumption of $RN_{j}^{k}$ can be calculated as follows:

(9) $$\begin{array}{ll} E_{cons}(RN_{j}^{k}) & =N_{in}\left(RN_{j}^{k}\right)\times E_{R}+N_{in}\left(RN_{j}^{k}\right)\times E_{T}\left(RN_{j}^{k},RN_{j}^{k+1}\right)\\ & =N_{in}\left(RN_{j}^{k}\right)\times \left(E_{R}+E_{T}\left(RN_{j}^{k},RN_{j}^{k+1}\right)\right)\\ & =N_{in}\left(RN_{j}^{k}\right)\times m\times \left(\gamma_{1}+\beta_{1}+\beta_{2}\times dis{\left(RN_{j}^{k},RN_{j}^{k+1}\right)}^{n}\right) \end{array}$$

where *m* is the size of data packet.

Let $minLf_{PTree_{i\;}}\;$ be the minimum lifetime of the routing tree *PTree_i_*. Therefore, our first objective is:

(10) $$\mathrm{Objective}\;1:\text{Maximize }minLf_{PTree_{i}}=\min\left\{lf\left(RN_{j}^{k}\right)|RN_{j}^{k}\in PTree_{i}\right\}$$

Using Equation (9) to substitute the $E_{cons}(RN_{j}^{k})$ in Equation (7), then,

(11) $$lf\left(RN_{j}^{k}\right)=E_{res}\left(RN_{j}^{k}\right)/E_{cons}\left(RN_{j}^{k}\right)=E_{res}\left(RN_{j}^{k}\right)/N_{in}\left(RN_{j}^{k}\right)\times m\times \left(\gamma_{1}+\beta_{1}+\beta_{2}\times dis{\left(RN_{j}^{k},RN_{j}^{k+1}\right)}^{n}\right)$$

It can be seen that, if *E_res_*($RN_{j}^{k}$) and *N_in_*($RN_{j}^{k}$) are fixed, then *lf*($RN_{j}^{k}$) is negatively correlated with dis($RN_{j}^{k}$, $RN_{j}^{k+1}$). Therefore, it can be deduced that relay node with minimum lifetime is the relay node with the longest transition distance. That is,

(12) $$minLf_{PTree_{i}}=RN_{j}^{k}=\arg\;\max dis\left(RN_{j}^{k},RN_{j}^{k+1}\right)\;|\forall j,1\leq j\leq N_{path}^{sink_{i}}\text{ and }\forall k,1\leq k\leq N_{Path_{j}}$$

where argmax*f*(*x*) returns the value of *x* that maximizes *f*(*x*).

Bigger $minLf_{PTree_{i\;}}\;$means smaller fitness value. Therefore, *fitness* is inversely proportional to the $minLf_{PTree_{i}}$, i.e.,

(13) $$fitness\;\propto \;1/minLf_{PTree_{i\;}}$$

where 0 < 1/$minLf_{PTree_{i\;}}$ ≤ 1.

$minLf_{PTree_{i\;}}$ is used to avoid node with lower residual energy being selected as sharing relay node whose load burden is heavy.

(2) Routing Path Length

In our network mode, the energy is mainly consumed to collect network statuses and forward data to sink. The unicast local flooding mechanism is adopted to save energy for collecting network statuses. It is well known that the energy consumption of sensor node in WSNs is subject to the transmission distance—shorter data dissemination paths lead to decreased energy consumption together with increased throughput and reliability, which also can be deduced from Equation (9). Therefore, our second objective is to minimize the length of the routing tree to minimize the forwarding data energy consumption. Let $Path_{j}\;$ be routing tree of $Agent_{i}$. Now, we calculate the total energy consumption of *PTree_i_* (i.e., $E_{cons}^{PTree_{i}}$). According to Equation (9), the energy consumption of each routing path $Path_{j}$ ($Path_{j}$ ⊆ $PTree_{i}$), i.e., $E_{cons}^{Path_{j}}\;$, can be calculated as follows:

(14) $$\begin{array}{ll} E_{cons}^{Path_{j}} & =\sum_{k=1}^{N_{Path_{j}}}E_{cons}\left(RN_{j}^{k}\right)=\sum_{k=1}^{N_{Path_{j}}}N_{indata}\left(RN_{j}^{k}\right)\times m\times \left(\gamma_{1}+\beta_{1}+\beta_{2}\times dis{\left(RN_{j}^{k},RN_{j}^{k+1}\right)}^{n}\right)\\ & =\sum_{k=1}^{N_{Path_{j}}}N_{in}\left(RN_{j}^{k}\right)\times m\times \left(\gamma_{1}+\beta_{1}\right)+\beta_{2}\times \sum_{k=1}^{N_{Path_{j}}}dis{\left(RN_{j}^{k},RN_{j}^{k+1}\right)}^{n} \end{array}$$

Therefore, $E_{cons}^{PTree_{i}}$ can be calculated as follow:

(15) $$E_{cons}^{PTree_{i}}=\sum_{j=1}^{N_{path}^{sink_{i}}}E_{cons}^{Path_{j}}=\sum_{j=1}^{N_{path}^{sink_{i}}}\sum_{k=1}^{N_{Path_{j}}}N_{indata}\left(RN_{j}^{k}\right)\times m\times \left(\gamma_{1}+\beta_{1}\right)+\beta_{2}\times \sum_{j=1}^{N_{path}^{sink_{i}}}\sum_{k=1}^{N_{Path_{j}}}dis{\left(RN_{j}^{k},RN_{j}^{k+1}\right)}^{n}$$

For each *Path_j_* assume that no packet is lost during routing data to agent, then *N_in_*($RN_{j}^{k}$) is the same for each relay node in *Path_j_* which can be considered as a constant value. Let *N_in_*($RN_{j}^{k}$) = *N_in_*. Then Equation (14) is improved as follows:

(16) $$E_{cons}^{Path}\left(Path_{j}\right)=N_{Path_{j}}\times N_{in}\times m\times \left(\gamma_{1}+\beta_{1}\right)+\beta_{2}\times \sum_{k=1}^{N_{Path_{j}}}dis{\left(RN_{j}^{k},RN_{j}^{k+1}\right)}^{n}$$

where $\sum_{i=1}^{N_{Path_{j}}}dis\left(RN_{j}^{k},RN_{j}^{k+1}\right)$ is length of the routing path $Path_{j}$. It is can be seen that $E_{cons}^{Path_{j}}$ is only positively correlated with routing path.

Therefore, to minimize $\;E_{cons}^{PTree_{i}}$, the total routing tree length *Len*(*PTree_i_*) needs to be minimized. That is, our second objective can be described as follows:

(17) $$\mathrm{Objective}\;2:\text{Minimize }Len\left(PTree_{i}\right)=\sum_{j=1}^{N_{path}^{sink_{i}}}\sum_{k=1}^{N_{Path_{j}}}dis\left(RN_{j}^{k},RN_{j}^{k+1}\right)$$

Smaller *Len* ($PTree_{i}$) is better. Therefore, *fitness* is proportional to the *Len* ($PTree_{i}$) i.e.,

(18) $$fitness\;\propto Len\;\left(PTree_{i}\right)$$

Now, we normalize *Len* (*PTree_i_*) by *allLen*, i.e.,

(19) $$Len\left(PTree_{i}\right)=\sum_{j=1}^{N_{path}^{sink_{i}}}\sum_{k=1}^{N_{Path_{j}}}dis{\left(RN_{j}^{k},RN_{j}^{k+1}\right)}^{n}/allLen$$

*allLen* can be calculated as follows:

(20) $$\begin{array}{l} allLen=\sum_{i=1}^{N_{V}}\sum_{j=1}^{N_{V}}dis(i,j)\times a_{ij}\;where\\ a_{ij}=\left\{ \begin{array}{l} 1\;if\;node_{j}\;within\;the\;communicaiton\;rang\;of\;node_{i},\;\forall i,j:1\leq i,j\leq N_{V}\\ 0\;Otherwise \end{array}\right. \end{array}$$

where *N_V_* is the number of candidate relay nodes. Therefore,

(21) $$Len\left(PTree_{i}\right)=\sum_{j=1}^{N_{path}^{sink_{i}}}\sum_{k=1}^{N_{Path_{j}}}dis{\left(RN_{j}^{k},RN_{j}^{k+1}\right)}^{n}/\sum_{i=1}^{N_{V}}\sum_{j=1}^{N_{V}}dis(i,j)\times a_{ij}$$

It can be deduced from Equation (21) that 0 < *Len* (*PTree_i_*) ≤ 1.

(3) Communication Delay

End-to-end delay is another important performance metric of MWSNs. Given a fixed channel bandwidth, less the delay, higher the throughout. Let $Delay\left(PTree_{i}\right)$ be the total communication delay of $PTree_{i}$. Then $Delay\left(PTree_{i}\right)$ can be calculated as following:

(22) $$Delay\left(PTree_{i}\right)=\sum_{RN_{j}^{k}\in PTree_{i}}delay\left(RN_{j}^{k}\right)$$

Therefore, our third objective is:

(23) $$\mathrm{Objective}\;3:\text{Minimize }Delay\left(PTree_{i}\right)=\sum_{RN_{j}^{k}\in PTree_{i}}delay\left(RN_{j}^{k}\right)$$

Clearly, smaller $Delay\left(PTree_{i}\right)\;$is better. Then our *fitness* is proportional to the $Delay\left(PTree_{i}\right)$ i.e.,

(24) $$fitness\;\propto Delay\left(PTree_{i}\right)$$

Similar with Equation (19), $Delay\left(PTree_{i}\right)$ also can be normalized as follows:

(25) $$Delay\left(PTree_{i}\right)=\sum_{RN_{j}^{k}\in PTree_{i}}delay\left(RN_{j}^{k}\right)/\sum_{node_{i}\in V}delay\left(node_{i}\right)$$

After normalizing the above three objectives, the final fitness function for $PTree_{i}\;$ is:

(26) $$fitness\left(PTree_{i}\right)=\omega_{1}\times minLf_{PTree_{i}}+\omega_{2}\times Len\left(PTree_{i}\right)+\omega_{3}\times Delay\left(PTree_{i}\right)$$

where *ω_1_*, *ω_2_* and *ω_3_* are three control parameters, 0 < *ω_i_* ≤ 1 and *ω_1_* + *ω_2_* + *ω_3_* = 1. In the paper, we give the same weight to them, that is, *ω_1_* = *ω_2_* = *ω_3_* = 0.33.

#### 5.2.2. Particles Representation

How to encode the routing tree is very critical. To make GMDPSO feasible for discrete MWSNs scenario, the position and velocity of particle in our protocol are redefined.

**Definition 1 (Position).**  *The position vector provides a routing tree. The position vector of ith particle is defined as X_i_ = {$x_{i}^{1}$, $x_{i}^{2}$, $x_{I}^{3}$, …, $x_{i}^{N^{\prime }}$}, where $N^{\prime \;}$= N − 1 is dimension of X_i_ which means the number of candidate relay nodes excluding the agent, the d component $x_{i}^{d}$ ∈ {0, N} is the sensor ID, which maps node_k_ (k = $x_{i}^{d}$) as the next relay node of node_d_. That is to say, $x_{i}^{d}$ indicates that node_d_ forwards data to node_k_*.

**Figure 4:**
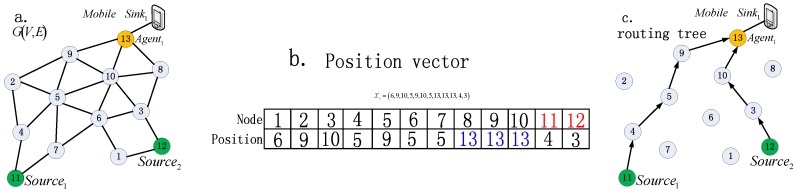
(**a**) Network topology; (**b**) Position vector encoded for (**a**); (**c**) Routing tree decoded from (**b**).

Notably, *X_i_* means a routing path tree that includes multi routing paths from multi source nodes to the same root node. Suppose mobile sink *Sink_i_* has *N_S_* source nodes, and then *X_i_* includes *N_S_* routing paths. A graphical illustration of particle representation can be seen in Figure 4.

**Illustration 1.**  *Consider mobile sink Sink_i_ masters a subnet of MWSN G (V, E) with 13 sensor nodes and one mobile node as shown in Figure 4a. V = {node_1_, node_2_, …, node_13_}. node_13_ is selected as agent, node_11_ and node_12_ are two source nodes, which means two routing paths will be built. The dimension of the particle position vector is $N^{\prime }$ = N − 1 = 13 − 1 = 12. As shown in Figure 4b. Particle i is encoded as X_i_ = {$x_{i}^{1}$, $x_{i}^{2}$, $x_{I}^{3}$, …, $x_{i}^{12}$} = {6, 9, 10, 5, 9, 5, 5, 13, 13, 13, 4, 3}, in X_i_, for example, $x_{I}^{11}$ = 4 indicates that source node node_11_ sends data packet to node_4_. A routing tree is constructed by decoding the encoded particle, in which each route path from a source node to sink can be built by appending relay nodes one by one till the agent node is selected as the end*.

As shown in Figure 4c, the path tree can be decoded as follows: firstly, the routing path of *Source_1_* (i.e., *node_11_*) is built as *Path_1_*: *Source_1_*→node_4_→node_5_→node9→*Agent_1_*.Then, routing path of *Source_2_* (i.e., *node_1__2_*) is built as *Path_2_*:*Source_2_*→node_3_→node_10_→*Agent_1_*, and at last, the whole routing tree can be constructed by combining *Path_1_* and *Path_2_*.

It can be seen from Figure 4 that our discrete position definition is straightforward and easy to decode and will lower the computational complexity, especially in the case of large-scale MWSNs, because the dimensions of the fitness function is equal to the size of candidate relay nodes collected by sink which is smaller than the entire MWSN size.

**Definition 2 (Velocity).**  *Velocity is a very crucial component in PSO, by working on the position vector, it guides a particle and determines whether it can reach its destination and by how fast it could. Our discrete velocity of particle i is defined as V_i_ = {$v_{i}^{1}$, $v_{i}^{2}$, …, $\;v_{i}^{N^{\prime }}$}, where $v_{i}^{j}$ ∈{0,1} is binary-coded, $v_{i}^{j}$ = 1 means that the corresponding element $x_{i}^{j}$ in X_i_ will be changed, otherwise, $x_{i}^{j}$ keeps its original state. $v_{i}^{j}$ and $x_{i}^{j}$ have the same dimension*.

In canonical PSO, velocity is used to learn knowledge from itself and swarm and finally leads the particle to a better position. In addition, a threshold *V_max_* is used to inhibit particles from flying out of the boundaries because there is a situation whereby when the speed of a particle is substantial. Unlike the continuous optimization, we have known that, in our discrete MWSNs scenarios, to compare two different routing paths from the same source node to the same agent, we only take care about whether their relay nodes are the same. Furthermore, we only need to compare the two relay nodes ID value in the same position of two different routing paths. There are only two results: is equal or no, therefore, the velocity can be encoded binary, and we defined 0 means two relay nodes are the same, 1 means they are different.

The first motivation of the velocity definition is to actually reflect the differences between two position vectors. The second motivation of the velocity definition is to prevent particles from flying away, because our velocity is binary-coded, we no longer need *V_max_* parameter.

#### 5.2.3. Particle Swarm Initialization

A good initialization mechanism can reduce the searching space to reach global optima faster and promote diversity. Conventional random initialization method for PSO based algorithm is not applicable for our algorithm. The main reason is that random sequence of edges usually results in invalid routing tree that does not terminate on the agent node or that have loops. Therefore, we need to design a more efficient initialization method for our protocol. Based on our particle representation for discrete MWSNs, the position vectors initialization focus on how to map the next relay node, in other words, how to select the next relay node for the current one, for example, how to map another node as the next relay node for current node node_d_. Our main idea to solve this problem is that the next relay node for the current one is randomly selected from its neighbors. The mapping is done as follows:

Let *Neig*(node_d_) = {node_1_, node_2_, …, node_K_} = {$x_{t}^{j}$|0 ≤ *j* ≤ $N_{V}^{\prime }$ & 0 < A[d, j] < ∞} be the neighbors of node_d_, and A is the adjacent matrix for *G* (*V, E*),Then,

(27) $$node_{k}=Index\left(Neig\left(node_{d}\right),n\right)，\;1\leq n\leq \left|Neig\left(node_{d}\right)\right|$$

where Index (*Neig*(node_d_), *n*) is an indexing function that indexes *nth* node of *Neig*(node_d_) as the next relay node, and *n* is a randomly generated uniformly distributed integer number. Table 2 shows the nodes and their neighbors. Besides, Table 1 also illustrates how the next relay node is chosen.

In order to reduce the randomicity and blindness of swarm initialization, and at the same time speed up the convergence of our algorithm, the position vectors are initialized in such way:

***Step 1*** Agent node is forced to be mapped as the relay node of its neighbor nodes (seeing the blue number in Figure 4b. In detail, firstly, position vector of *i* particle is empty, that is, *Xi* = (0, 0, 0, 0, 0, 0, 0, 0, 0, 0, 0, 0). Then assign agent node (i.e., node_13_) to its neighbor nodes, as shown in Table 2, then *Xi* = ({0, 0, 0, 0, 0, 0, 0, 13, 13, 13, 0, 0}. Marker ‘-’ in Table 2 means that the relay node of the corresponding node is specified directly instead of choosing from its neighbors using Equation (27).

***Step 2*** Maps agent’s neighbors to be relay node of the agent’s neighbor’s neighbor. Notably, if some of the agent’s neighbors have the same neighbor, which cause more than one node will be mapped as the relay node of the same neighbor at the same time, and then we chose the nearest neighbor as the relay node. For example, as shown in Table 2, the neighbor of the agent (i.e., node_13_) is *Neig* (node_13_) = {node_8_, node_9_, node_10_}, then node_13_ is the common next relay node of *Neig* (node_13_). Next, we assign node_8_, node_9_, and node_10_ to their neighbors respectively. *Neig*(node_8_)∪*Neig*(node_9_)∪*Neig*(node_10_) = {node_2_, node_3_, node_5_, node_6_}, which means that node_8_, node_9_, and node_10_ should be assigned to node_2_, node_3_, node_5_ and node_6_ ,that is to say, the third column of Table 2 (i.e., column n) of the corresponding nodes, i.e., node_8_, node_9_, node_10_, node_2_, node_3_, node_5_ and node_6_ should be marked with ‘-’. It is easy to be seen form Figure 4a that *Neig* (node_2_) = node_9_, *Neig* (node_6_) = node_10_. For node_5_, because *Neig* (node_9_)∩*Neig*(node_10_) = {node_5_}, which means that node_9_ can be chosen as the next relay node for node_5_, and so do node_10_. We chose node_9_ as the relay node of node_5_ with assumption that node_9_ is the nearest neighbor of node_5_. For node_6_, we choose node_10_ as its next relay node in the same way. After finished *Step 2*, then the position vector *Xi* will look as following: *Xi* = {0, 9, 10, 0, 9, 10, 0, 13, 13, 13, 0, 0}.

***Step 3*** The relay nodes of remaining nodes are mapped by randomly choosing a node from their neighbor. For example, for *Source_1_* (i.e., node_11_), let random *n* = 1, then its first neighbor is chosen as the next relay node.

***Step 4*** Finally, after finished the above three initiation steps, position vector *Xi* is initiated as *Xi* = {6, 9, 10, 5, 9, 10, 5, 13, 13, 13, 4, 3}.

It is worth noting that the invalid routing tree, in which contains one or more invalid routing path (that does not terminate on the agent node or that have loops), will be punished with a very high fitness.

The velocity vectors are initialized as all-zero vectors. The *P_best_* vectors are initialized in the same manner as the position vectors, and the vector is set as the best position vector in the original population.

Comparing to the conventional random initialization, the advantages of our initiation method are follows:

(1) Since node can and only can select its next relay node from its neighbors based on the network topology of *G*(*V, E*), our method takes advantage of this feature to reduce the vast search space significantly.
(2) In our method, agent node and its neighbors are mapped firstly in ***step1*** and ***step3***, and this reduces the likelihood of invalid routing path. In addition, it further reduces search space and drives particle to find its personal best position faster. That is, it speeds the algorithm convergence.

#### 5.2.4. Velocity and Position Update

In canonical PSO, velocity gives a particle the moving direction and tendency. After updating its velocity, the particle builds its new position using the new velocity. However, in the proposed algorithm, the particle position and velocity vectors have been redefined in a discrete integer form, and thus, the mathematical updating rules in the canonical continuous PSO no longer fit the discrete case. In order to meet the requirements of building routing tree in discrete MWSNs, the particle’s velocity and position updating rules have been redefined as follows:

(28) $$V_{i}\left(t+1\right)=w\times V_{i}\left(t\right)⊕c_{1}\times r_{1}\times \left(pbest_{i}⊖X_{i}\left(t\right)\right)+c_{2}\times r_{2}\times \left(Gbest⊖X_{i}\left(t\right)\right)$$

(29) $$\;X_{i}\left(t+1\right)=X_{i}\left(t\right)⊗V_{i}\left(t\right)$$

where *w* is the dimensional inertial weight vector, *c_1_* and *c_2_* are two $N_{V}^{\prime }$ dimensional cognitive and social components, *r_1_* and *r_2_* are two $N_{V}^{\prime }$ dimensional random vectors in rang [0,1]. Equation (28) is used to update the old velocity, and Equation (29) is the position updating rule. It can be observed that the new updating rules take the same form in canonical PSO but different the key operator. The following will detail our new operators in discrete PSO.

**Definition 3 (Position Operator).**  *⊝. Position ⊝ Position builds a velocity vector. Assume that we are given two position vector X_i_ = {$x_{i}^{1}$, $x_{i}^{2}$, $x_{i}^{3}$ … $x_{i}^{N^{\prime }}$} and X_j_ = {$x_{j}^{1}$, $x_{j}^{2}$, $x_{j}^{3}$, …, $x_{j}^{N^{\prime }}$}, X_i_ ⊝ X_j_ = V = {v_1_, v_2_, …, $v_{N^{\prime }}$}, the element v_d_ is defined as:*

(30) $$v_{d}=\left\{ \begin{array}{l} 0\;if\;x_{i}^{k}=\;x_{j}^{k}\\ 1\;otherwise \end{array}\right.$$

Definition 3 is inspired from the following two aspects: 

First, it is well known that, in canonical PSO, a particle adjusts its velocity by learning from its old velocity (*V_i_*(*t*)), personal best position (*Pbest_i_*-*X_i_*(*t*)) and the swarm global best position (*Gbest*-*X_i_*(*t*)). It can be seen that the learning process is actually a comparison between the positions, that is to say, two position vectors should generate a velocity vector. Second, two position vectors represent two different routing trees. The defined ⊝ operation actually is used to find which relay nodes are different between these two routing paths, and these differences will give a particle the fly direction.

**Definition 4 (Velocity Operator).**  *⊕. Velocity ⊕ Velocity is also a velocity vector. Given two velocity vectors V_1_ = {$v_{1}^{1}$, $v_{1}^{2}$, …, $v_{1}^{N^{\prime }}$} and V_2_ = {$v_{2}^{1}$, $v_{2}^{2}$, …, $v_{2}^{N^{\prime }}$}, then V_3_ = V_1_⊕ V_2_ = {$v_{3}^{1}$, $v_{3}^{2}$, …, $v_{3}^{N^{\prime }}$}. $v_{3}^{i}$ can be compute as follows:*

(31) $$v_{3}^{k}=\left\{ \begin{array}{l} 1\;condition\;1:if\;v_{1}^{k}+v_{2}^{k}\geq 1\\ 0\;condition\;2:otherwise \end{array}\right.$$

Suppose *V_1_* = *Pbest_i_* ⊝*X_i_*(*t*) and *V_2_* = *Gbest* ⊝*X_i_*(*t*), then, *condition1* means that once the *kth* relay node in particle *i* (i.e., $v_{1}^{k}$ = 1) needs to be changed, which is decided by its own knowledge (i.e., $v_{1}^{k}$ = 1) or by swarm knowledge (i.e., $v_{2}^{k}$ = 1) or both of them (i.e., $v_{1}^{k}$ = 1 & $v_{2}^{k}$ = 1), it must be changed finally. The definition of the operator ⊕ is easy to understand and easy to perform. Moreover, it can ensure binary-coded consistency for velocity, which is easier for the position to work with.

**Definition 5 (Coefficient × Velocity).**  *Coefficient × Velocity is still velocity vector. Given the Coefficient ω and the velocity vector V_1_ = $v_{1}^{1}$, $v_{1}^{2}$, …, $v_{1}^{N^{\prime }}$}, then V_2_ = ω × V_1_ = {$v_{2}^{1}$, $v_{2}^{2}$, …, $v_{2}^{N^{\prime }}$}. Where $v_{2}^{i}$ can be compute as follows:*

(32) $$v_{3}^{i}=\left\{ \begin{array}{l} 1\;condition\;1:if\;v_{1}^{i}=1\\ 1\;condition\;2:if\;v_{1}^{i}=0\text{ \& }\omega \geq 0.5\\ 0\;condition\;3:if\;v_{1}^{i}=0\text{ \& }\omega <0.5 \end{array}\right.$$

*condition1 means that if a relay node needs to be changed , then change it without regard to the ω. Condition2 and condition3 are conditions for mutation operation of particle, which means that although a relay node does not need to be changed(i.e., $v_{1}^{i}$ = 0), it still maybe be changed in a mutation probability (i.e., 0.5). Mutation operation of particle can promote the diversity of particles and avoid falling into the local optimum*.

**Definition 6 (Position Updating Operato**r) *⊗. ⊗ is very important component in our update rules, which is used by particle to update its position with its new velocity. Position ⊗ Velocity generates a new position. A good design for operator ⊗ should drive a particle to a better position. Given an old position X_t_ = {$x_{t}^{1}$, $x_{t}^{2}$, …, $x_{2}^{N^{\prime }}$} and a new velocity V_t+1_ = {$v_{t+1}^{1}$, $v_{t+1}^{2}$, …, $v_{t+1}^{N^{\prime }}$}, then the new position X_t+1_ = X_t_ ⊗ V_t+1_ = {$x_{t+1}^{1}$, $x_{t+1}^{2}$, …, $v_{t+2}^{N^{\prime }}$}, the element $x_{t+1}^{i}$ of X_t+1_ can be computed as follows:*

(33) $$x_{t+1}^{i}=\left\{ \begin{array}{l} bestRN_{i}\;if\;v_{t+1}^{i}=1\text{ \& }x_{t}^{i}\in Path_{j}^{t+1}\\ \;x_{t}^{i}\;otherwise \end{array}\right.$$

*where $x_{t}^{j}$ ∈$Path_{j}^{t+1}$ means that the old node $x_{t}^{j}$ is selected as a relay node in the $Path_{j}^{t+1}$ in X_t+1_, in other words, if $x_{t}^{j}$ is not a relay node, it need not to be changed. Notably, X_i_ means a routing path tree that contains N_S_ routing path branches, and Path_j_ is only one routing path branch of the routing path tree. Therefore, only these old nodes will be changed that satisfies the following conditions simultaneously: they are chosen as the relay nodes and their corresponding velocities are set to 1, i.e. $v_{t+1}^{i}=1\text{ \& }x_{t}^{i}\in Path_{j}^{t+1}$. bestRN_i_ is the node ID which can be calculated by:*

(34) $$bestRN_{i}\;=\arg\min_{k}\varphi \left(i,k|k\in Neig\left(i\right)\right)$$

*argmin_k_ f(k) returns the value of k that minimizes f(k). φ is calculated using the following equation:*

(35) $$\varphi \left(i,j|k\in Neig\left(i\right)\right)\;=fit\left(Path_{j}\left(i\to k\right)\right)$$

*where i→k means that node_k_ is chosen as the next relay node of node_i_. fit(x) is the fitness function (i.e., Equation (26)), and fit(Path_j_(i→k)) means that we only calculate the fitness value of the two adjacent relay nodes in Path_j_. Similar to greedy forwarding mechanism, Equation (35) means we choose the best next relay node for the current node from all its neighbors. However, unlike greedy mechanism that only chooses the nearest neighbor as the best next relay node, our approach chooses the neighbor node, which achieves the best balance of lifetime, distance and delay as the best next relay node by using our fitness function. Therefore, with the beginning from Source_j_. Path_j_ can be built by selecting the best relay node one after one until the agent node is chosen, and Path_j_ that is built in this way is the optimal routing path. For example, let first bestRN_1_ be $RN_{j}^{1}$, then $Path_{j}^{1}=\left(Source_{j}\to RN_{j}^{1}\right)$ is the current optimal routing path. Let $Path_{j}^{2}$ be the bestRN_2_ of $RN_{j}^{1}$, $Path_{j}^{2}=\left(RN_{j}^{1}\to RN_{j}^{2}\right)$ is the current optimal routing path. Then,*

(36) $$\begin{array}{l} Path_{j}^{1}=\left(Source_{j}\to RN_{j}^{1}\right)\\ Path_{j}^{2}=\left(RN_{j}^{1}\to RN_{j}^{2}\right) \end{array}\}\Rightarrow Path_{j}=\left(Source_{j}\to RN_{j}^{1}\to RN_{j}^{2}\right)$$

*where Path_j_ is also the optimal routing. Using the same way, the other routing branches of X_t_ can be built. Next, the fitness of the X_t_ can be calculated using Equation (26).*

This search mechanism of *bestRN_i_* (i.e., Equation (34)) actually act as our particle searching strategy in GMDPSO, in which a particle update its position by selecting the next relay node that can generate the largest decrement of the fitness value, so it can be regard as a greedy local search strategy.

Our motivation of searching *bestRN_i_* in this way is based on the divide-conquer strategy, in order to build an optimal routing tree of *X_t_*, we firstly build each optimal routing path branch of *X_i_* respectively. When all routing path branches are built completely, then, the whole routing path tree is finished. Similarly, in order to build the optimal routing path branch *Path_j_*, we choose the best next relay node for the previous relay node one after one, until the whole *Pathj* is completed.

**Figure 5:**
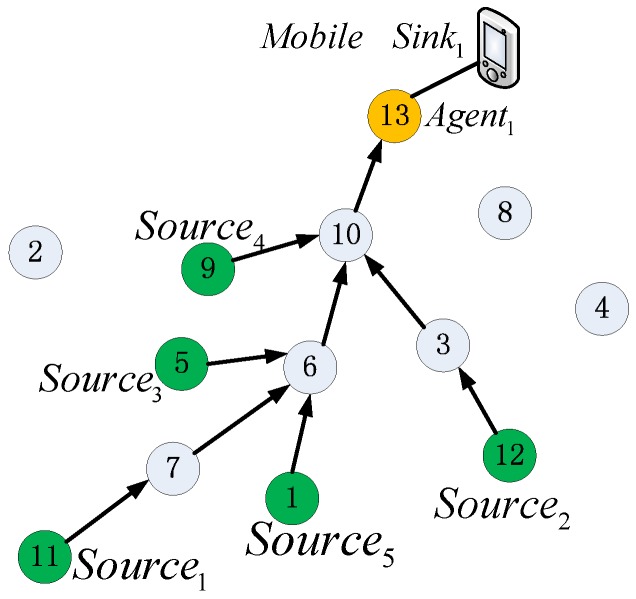
Multi routing paths share some relay nodes.

However, Equation (34) is only used to build the single optimal routing path branch, rather than build the routing path tree (i.e., *X_i_*). Because when we choose the best relay node for the current node of *Path_j_*, we do not consider the effect of the other routing path branch. Therefore, this method may neglect such a case, each routing branch is optimal, but the whole routing tree are weak due to the intersection relay nodes of multi routing paths. In other words, optimal routing path branches do not mean the optimal routing tree. As shown in Figure 5, many source nodes send their data packets to same next relay node, and these relay nodes will consume more energy to forward more packets. For example, *node_6_* need forward data packets of *Source_1_*, *Source_2_* and *Source_3_* to *node_10_*, while, *node_10_* needs forward data packets of *Source_1_*, *Source_2_*, *Source_3_*, *Source_4_* and *Source_5_* to *Agent_1_*. The worst result is that these sharing relay nodes will die quickly because they deplete their energy of forwarding too many packets. This will lead to the expensive routing recover.

Therefore, after all of individual optimal routing path branches are built, the corresponding routing tree *X_i_* should be evaluated using fitness function.

From the above description of our new GMDPSO algorithm, the proposed algorithm has the following features: (1) with a concise framework; (2) the newly defined particle position and velocity are direct and easy to decode; and (3) redefined updating rules based on the new operators are easy to realize and will significantly reduce the computational complexity, especially for large-scale WSNs.

## 6. The Redesigned GMDPSO Seems to be Very Suitable for Solving Routing Problem in MWSNs6. Simulations and Results

In this section, we test our protocol against several well-known protocols: ECPSOAR, IAR, and TTDD, which all can deal with routing problem of multi mobile sinks. The performance is compared in terms of the following metrics: average packet delivery ratio (PDR, measured as the average number of successfully delivered packets versus required packets per round), average end-to-end delay (EED) [29], and average energy consumption ratio (ECR, measured as the average energy consumption from source to sink versus the initial energy per round). All simulations are performed using MATLAB R2012b on Windows 7 with Intel core i5-2520M Dual-Core CPU (2.50 GHz) and 8 G RAM. For ease of description for the comparison results of the above metrics, we define another notation, which is called metric comparative advantage for our new protocol as defined below:

(37) $$\begin{array}{l} Metric\;comparative\;advantage=\\ \;\frac{\left(Metric\;of\;GMDPSO\;-\;Metric\;of\;other\;protocol\right)}{Metric\;of\;other\;protocol}\times 100 \end{array}$$

For example, PDR comparative advantage to ECSPOAR can be calculated as follow:

(38) $$\begin{array}{l} PDR\;comparative\;advantage\;to\;ECPSOAR=\\ \;\frac{\left(PDR\;of\;GMDPSO\;-\;PDR\;of\;ECPSOAR\right)}{PDR\;of\;ECPSOAR}\times 100 \end{array}$$

### 6.1. Simulation Setting

Simulations are performed on the MWSN, which consists of diverse number of homogenous sensor nodes ranging from 50 to 450. Each sensor node is assumed to have initial energy of 120 J and the mobile sink is assumed to have sufficient energy and cannot be fault.

To build a level playing field, the characteristics of the networks and communication models are configured as illustrated in [10] and as shown in Table 3. The extra PSO parameters used for ECPSOA are fixed to: particle updating energy consumption *E_PU_* = 80 *pJ* the endocrine selection energy consumption *E_ES_* = 50 *pJ* at per iteration, function dimension *D* = 30, division factory *k* = 6, and maximum iteration *P_Gen_* = 800 .The extra PSO parameters used for GMDPSO are fixed to: ω = 0.7968, c1 = c2 = 1.4926 In addition, the population size of ECPSOA and DPSORR is set to be 60; both of the two algorithms are run for maximum of 800.

For the weight sum approach, in our proposed algorithms, we give equal weight to each sub-objective. That to say, we set *w_1_* = *w_2_* = *w_3_* = 0.33.

### 6.2. Results and Analysis

#### 6.2.1. Performance of GMDPSO

First, we compare the performance of the proposed GMDPSO with ECPSOA, standard PSO [30] and the CPSOA [31]. In order to ensure a fair comparison, we configure these tree algorithms based on the same fitness function in Equation (26) with 450 sensor nodes, all simulation parameters are set to the same value, and the iterated generation for three protocols is fixed to 300.

**Figure 6:**
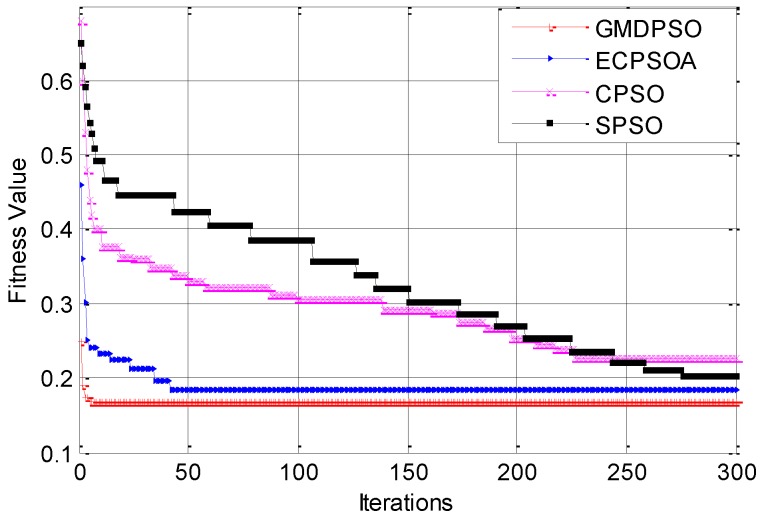
Compare of convergence.

The test result is shown in Figure 6. It can be seen that: GMDPSO outperforms the other PSOAs in term of convergence rate and the minimum fitness value. This is mainly due to the greedy search strategy based on the MWSNs topology, which avoid the blind search of the particle; another reason is that the memory mechanism reduces the repeated and invalid searching. It also can be seen that GMDPSO has the best initial fitness value, which is achieved by our special particle initial mechanism.

#### 6.2.2. Packet Delivery Ratio

**Figure 7:**
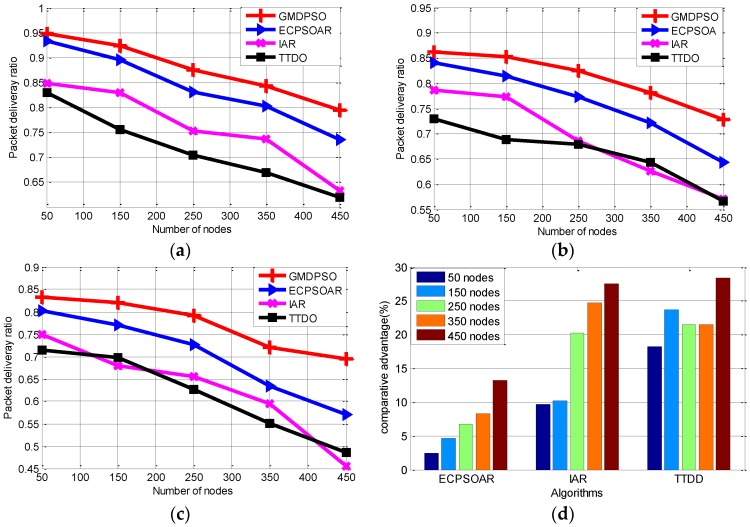
Average packet delivery ratio with respect to different node failure probabilities. (**a**) When the node failure probability is 0.01; (**b**) When the node failure probability is 0.02; (**c**) When the node failure probability is 0.04; (**d**) Average PDR comparative advantage.

A routing protocol’s reliability depends on PDR to the sink. Here, we run these protocols for comparing average PDR. Figure 7 and Table 4 show the comparison results when the speed of sinks is 5 m/s. Figure 7 shows that our protocol is more reliable and robust. In detail, firstly, average PDR of all protocols are decreasing as the number of nodes increases; however, our protocol still outperforms the others and the advantage (i.e., Equation (17)) is becoming more and more obvious as the number of sensor nodes increase in Figure 7d. Secondly, PDR reduces with the increase of the node failure probability (Figure 7a–c). Still, our protocol can deliver more packets than the other protocols with the same network size. Thirdly, with each same $P_{fault}$,*_t_*, our protocol always keeps the maximum average PDR (Figure 7a–c), which means that our protocol can find a better solution than others respect to different sensor nodes. Meanwhile, Table 4 illustrated that, with each same sensor node, our protocol keeps the maximum average PDR, which means that our protocol can find a better solution than others respect to different $P_{fault}$. That is to say, no matter with different sensor nodes or different node failure probability, our protocol can find the best path tree among the 4 protocols. Therefore, based on the above two results, we conclude that our protocol is more robust in PDR.

Our protocol improved the average PDR noticeably due to the following three reasons: The first reason is, the temporary path (TP) is designed to continue the old data packets transmission when sink moves away the old agent, which can avoid the packets loss before the new optimal routing path coming into services. The second reason is: our protocol can quickly locate the failure node and quickly build an alternative optimal path to recover the broken path link by its fast convergence feature and without any flooding when relay nodes fail, which also can reduce the data packets loss. The third reason is: the proposed GMDPSO can build better alternative routing path than others due to its ability of achieving global optimum, such as shorter total transmission distance and small communication delays, which can enhance the communication reliability and PDR. Moreover, our fitness function minimizes the energy consumption of the relay nodes to reduce the premature death probability of the relay node, which also can reduce the data packets loss due to the broken routing path.

#### 6.2.3. End-to-End Delay

**Figure 8:**
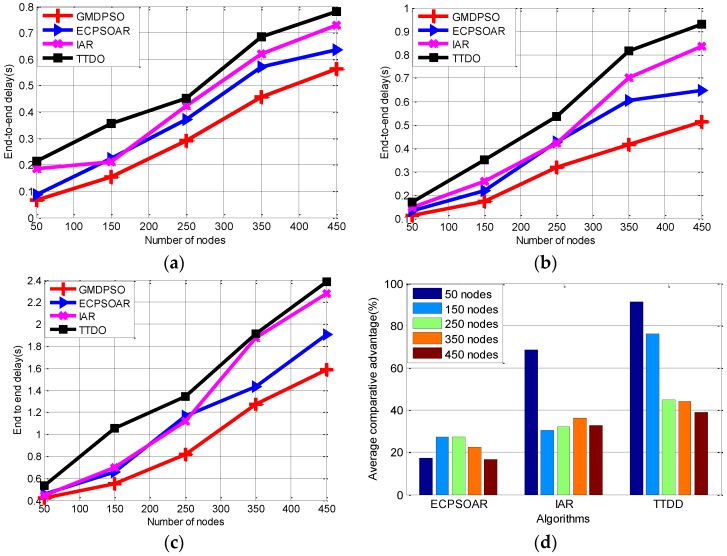
Average end-to-end delay with respect to different node failure probabilities. (**a**) When the node failure probability is 0.01; (**b**) When the node failure probability is 0.02; (**c**) When the node failure probability is 0.04; (**d**) Average EED comparative advantage.

Next, we compare the average EED of the proposed protocol on the same experiment environment as Section 6.2.1. Figure 8 and Table 5 show the comparison result. It can be observed from Figure 8 that the proposed protocol has smaller end-to-end delay than the existing protocols.

More specifically, firstly, with the increase of the number of nodes, EED of all these protocols is increased. Again, our protocol performs best. This is because our protocol adopts the GMDPSO algorithm, whose better performance in reaching the global optimum allows it to build the optimal routing tree with shorter transmission distance. Furthermore, its faster convergence feature make it can build the optimal routing tree more quickly. Moreover, by using our neighbor table, the routing information is stored in each node to improve the speed of route establishment. All these advantages of GMDPSO can help to decrease the end-to-end delay. It is worthy to note that, as illustrated in Figure 8d, the average EED comparative advantage (i.e., Equation (17)) decreases as the number of sensor nodes increases, this is because our greedy search rule spends more time to select the best relay nodes from larger scale nodes to build the optimal routing tree. Nevertheless, the fast response of routing recovery and less communication control overhead by our unicast flooding mechanism also make our protocol end-to-end delay lower than the other protocols. Secondly, the end-to-end delay increases with the increase of the node failure probability by comparing Figure 8a–c, as the less stable topology causes more route recovery operation, which consumes more time for the protocols to maintain the network and prolong the delay. However, our protocol can still achieve the optimal delay; this is because we have designed the quick routing recovery mechanism for failure relay nodes. Thirdly, similar to average PDR, it can be observed from Figure 8a–c and Table 5 that our protocol achieves best average EED with respect to different sensor nodes and different $P_{fault}$, which means that our protocol can find a better solution than others. That to say, our protocol keeps better robustness of EED, this is because that the GMDPSO adopted in our protocol can also build the global optimum routing tree in different network size, however, others maybe build different suboptimal routing solutions for different network sizes, which increase the volatility of delay. Moreover, once the existing routing path is broken due to failure nodes, an alternative optimal path can be quickly established for the source node in our protocol. In addition, the other failure nodes (i.e., not the relay nodes) never be selected as the relay nodes due to our fitness function.

#### 6.2.4. Energy Consumption

**Figure 9:**
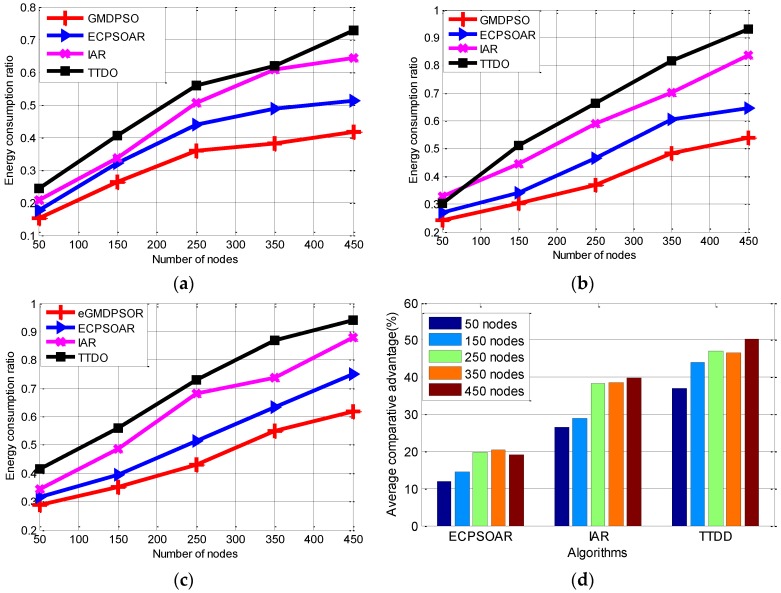
Average energy consumption ratio (ECR) with respect to different speeds of mobile sinks. (**a**) When the moving speed of sinks is 5 m/s; (**b**) When the moving speed of sinks is 10 m/s; (**c**) When the moving speed of sinks is 20 m/s; (**d**) Average ECR comparative advantage.

Although mobile sink protocol can alleviate hotspots implicitly by changing the possible high energy consumption zones around the sinks as the sinks move. However, these operations may cause the overall energy consumption in the network to increase. Now, we compare the average ECR of these protocols, which is used to measure the influence of node failure probability and mobile sink speed to the network. Simulations are performed on different network sizes with different sink speeds (speed of 5 m/s, 10 m/s and 20 m/s). Here, the node failure probability is set to 0.1. Figure 9 and Table 6 illustrate that the energy consumption of our protocol is the smallest among these protocols, and the comparative advantage (i.e., Equation (17)) becomes larger as the number of sensor nodes increases in Figure 9d. It worth to note that the average ECR of all these protocols increases as the mobile sinks move faster, because the change of the frequent topology results in frequent routing recovery which introduces heavier communication and energy overhead due to flooding operation. In this situation, our protocol can still consume less energy than the other protocols with the same network size. Besides, similar to average PDR, we can also conclude that our protocol achieves better robustness of EED than others from Figure 9a–c and Table 6.

Our protocol outperforms others in term of ECR. The main reasons are as follows: the improved greedy forwarding mechanism is used ensure each routing branch has the mining energy consumption by selecting the relay node with optimized QoS parameters (energy, delay, energy consumption and so on). In addition, the unicast local flooding mechanism reduces the communication overhead in the network, which can minimize and indirectly reduce energy consumption.

In summary, the overall performance of our protocol outperforms LEACH, SEP, ERP and TPSO-CR in terms of the PDR, EED and EDR, while maintaining the best robust.

## 7. Conclusions

In this paper, the routing of MWSNs is formulated as an optimization problem and we employ PSO to design an efficient routing protocol to achieve higher energy efficiency and lower communication delay. However, conventional PSO was originally designed for continuous optimization problems, which limits its application in discrete optimization domains. In addition, conventional PSO suffers from the curse of dimensionality, i.e., its performance deteriorates quickly as the dimensionality of the search space increases exponentially. To address these problems, we design a novel GMDPSO to build the optimal route tree. In GMDPSO, we first deduced a new more suitable fitness function, then redefined the particle position and velocity in a discrete form and subsequently redesigned the particle update rules based on the network topology; consequently a discrete PSO framework was established. When applying the proposed discrete PSO framework to solve the mobile sink route problem, to alleviate prematurity, a greedy local search based method was specially designed for the particle position update rule by improving the greedy forwarding mechanism. Simulations demonstrated that the proposed protocol is effective and promising.

## Figures and Tables

**Table 1:** Neighbor table.

self_ID	neighbor_ID	Dis	isRelay
7	3	20	1
7	9	15	0

**Table 2:** Next relay node selection for position initiation.

node_d_	Neig(node_d_)	n	NextRelay(node_d_)
node_1_	{node_3_, node_6_, node_12_}	2	node_6_
node_2_	{node_4_, node_5_, node_9_}	-	node_9_
node_3_	{node_6_, node_8_, node_10_, node_12_}	-	node_10_
node_4_	{node_2_, node_5_, node_7_, node_11_}	2	node_5_
node_5_	{node_2_, node_4_, node_6_, node_7_, node_9_, node_10_}	-	node_9_
node_6_	{node_1_, node_3_, node_5_, node_7_, node_10_}	-	node_10_
node_7_	{node_4_, node_5_, node_6_, node_11_}	2	node_5_
node_8_	{node_3_, node_10_, node_13_}	-	node_13_
node_9_	{node_2_, node_5_, node_10_, node_13_}	-	node_13_
node_10_	{node_3_, node_5_, node_6_, node_8_, node_9_, node_13_}	-	node_13_
node_11_	{node_4_, node_7_}	1	node_4_
node_12_	{node_1_, node_3_}	2	node_3_
node_13_	{node_8_, node_9_, node_10_, sink}		sink

Blue color means the corresponding node are mapped in ***step1***. Green color means the corresponding node are mapped in ***step 2***.Black color means the corresponding node are mapped in ***step 3***.

**Table 3:** Simulation parameters.

Parameter	Value	Parameter	Value
Area	5000 × 5000 m^2^	Packet size	1 KB
Sensor nodes	50, 150, 250, 350, 450	Deliver packets rate	20 per round
Mobile sinks	5	Simulation iterations number	200
Initial energy of nodes	120 J	*α_1_*	60 nj/bit
communication rang	600 m	*β_1_*	45 nj/bit
sensing rang *R*	300 m	*β_2_*	10 nj/bit
Speed of mobile *v_sink_*	5 m/s, 10 m/s, 20 m/s	*γ_1_*	135 nj/bit
*P_fault_*	0.1, 0.2, 0.4	Channel attenuation *n*	2

**Table 4:** Average packet delivery ratio (PDR) with respect to different $P_{fault}$.

Algorithms	150 Sensor Nodes			350 Sensor Nodes			450 Sensor Nodes		
	$P_{fault}$.01	$P_{fault}$.02	$P_{fault}$.04	$P_{fault}$.01	$P_{fault}$.02	$P_{fault}$.04	$P_{fault}$.01	$P_{fault}$.02	$P_{fault}$.04
GMDPSO	0.924	0.852	0.821	0.843	0.781	0.721	0.794	0.728	0.694
ECPSOAR	0.896	0.814	0.771	0.803	0.721	0.634	0.736	0.643	0.571
IAR	0.829	0.773	0.679	0.737	0.626	0.595	0.633	0.571	0.456
TTDD	0.755	0.6890	0.698	0.670	0.643	0.551	0.620	0.567	0.486

**Table 5:** Average end-to-end delay (EED) with respect to different $P_{fault}$.

Algorithms	150 Sensor Nodes			350 Sensor Nodes			450 Sensor Nodes		
	$P_{fault}$.01	$P_{fault}$.02	$P_{fault}$.04	$P_{fault}$.01	$P_{fault}$.02	$P_{fault}$.04	$P_{fault}$.01	$P_{fault}$.02	$P_{fault}$.04
GMDPSO	0.155	0.173	0.551	0.457	0.417	1.273	0.563	0.513	1.585
ECPSOAR	0.223	0.220	0.659	0.571	0.605	1.431	0.635	0.646	1.908
IAR	0.212	0.260	0.700	0.620	0.702	1.878	0.729	0.835	2.278
TTDD	0.357	0.349	1.056	0.684	0.815	1.911	0.780	0.929	2.383

**Table 6:** Average ECR with respect to different $V_{Sink}$.

Algorithms	150 Sensor Nodes			350 Sensor Nodes			450 Sensor Nodes		
	$V_{Sink}$5	$V_{Sink}$10	$V_{Sink}$20	$V_{Sink}$5	$V_{Sink}$10	$V_{Sink}$20	$V_{Sink}$5	$V_{Sink}$10	$V_{Sink}$20
GMDPSO	0.264	0.302	0.352	0.382	0.482	0.549	0.416	0.539	0.618
ECPSO	0.321	0.341	0.395	0.487	0.605	0.634	0.512	0.646	0.749
IAR	0.336	0.445	0.486	0.607	0.702	0.737	0.643	0.835	0.880
TTDS	0.405	0.512	0.560	0.618	0.815	0.869	0.728	0.929	0.941

## References

[1] Rault T., Bouabdallah A., Challal Y. (2014). Energy efficiency in wireless sensor networks: A top-down survey. Comput. Netw..

[2] Jaichandran R., Irudhayaraj A.A., Raja J.E. Effective Strategies and Optimal Solutions for Hot Spot Problem in Wireless Sensor Networks (WSN). Proceedings of the 2010 10th International Conference on Information Sciences Signal Processing and their Applications (ISSPA).

[3] Francesco M.D., Das S.K., Anastasi G. (2011). Data Collection in Wireless Sensor Networks with Mobile Elements: A Survey. ACM Trans. Sens. Netw..

[4] Liang W., Luo J., Xu X. Prolonging Network Lifetime via a Controlled Mobile Sink in Wireless Sensor Networks. Proceedings of the 2010 IEEE Global Telecommunications Conference (GLOBECOM 2010).

[5] Nazir B., Hasbullah H. Mobile Sink based Routing Protocol (MSRP) for Prolonging Network Lifetime in Clustered Wireless Sensor Network. Proceedings of the 2010 International Conference on Computer Applications and Industrial Electronics (ICCAIE).

[6] Oh S., Yim Y., Lee J., Park H., Kim S.H. Non-Geographical Shortest Path Data Dissemination for Mobile Sinks in Wireless Sensor Networks. Proceedings of the 2011 IEEE Vehicular Technology Conference (VTC Fall).

[7] Liu X., Zhao H., Yang X., Li X. (2013). SinkTrail: A Proactive Data Reporting Protocol for Wireless Sensor Networks. IEEE Trans. Comput..

[8] Shi G., Zheng J., Yang J., Zhao Z. (2012). Double-Blind Data Discovery Using Double Cross for Large-Scale Wireless Sensor Networks With Mobile Sinks. IEEE Trans. Veh. Technol..

[9] Tunca C., Isik S., Donmez M.Y., Ersoy C. (2014). Distributed Mobile Sink Routing for Wireless Sensor Networks: A Survey. IEEE Commun. Surv. Tutor..

[10] Hu Y.F., Ding Y.S., Ren L.H., Hao K.R., Han H. (2015). An endocrine cooperative particle swarm optimization algorithm for routing recovery problem of wireless sensor networks with multiple mobile sinks. Inf. Sci..

[11] Hu Y.F., Wu X.M., Wang F.Q., Han H. A particle swarm algorithm based routing recovery method for mobile sink wireless sensor networks. Proceedings of the 26th Chinese Control and Decision Conference (2014 CCDC).

[12] Hu Y.F., Wu X.M., Wang F.Q., Liu X.Z., Han H. A novel routing recovery strategy based on particle swarm algorithm for wireless sensor networks with multiple mobile sinks. Proceedings of the 2014 13th International Conference on Control Automation Robotics & Vision (ICARCV).

[13] Chengetanai G., Reilly G.B.O. Review of swarm intelligence routing algorithms in wireless mobile ad hoc networks. Proceedings of the 2015 IEEE 9th International Conference on Intelligent Systems and Control (ISCO).

[14] Tang D., Cai Y., Zhao J., Xue Y. (2014). A quantum-behaved particle swarm optimization with memetic algorithm and memory for continuous non-linear large scale problems. Inf. Sci..

[15] Luo H., Ye F., Cheng J., Lu S., Zhang L. (2005). TTDD: Two-Tier Data Dissemination in Large-Scale Wireless Sensor Networks. Wirel. Netw..

[16] Kweon K., Ghim H., Hong J., Yoon H. Grid-Based Energy-Efficient Routing from Multiple Sources to Multiple Mobile Sinks in Wireless Sensor Networks. Proceedings of the ISWPC 2009 4th International Symposium on Wireless Pervasive Computing.

[17] Minhan S., Chunum K., Choo H. Hexagonal path data dissemination for energy efficiency in wireless sensor networks. Proceedings of the 2009 International Conference on Information Networking.

[18] Kim H.S., Abdelzaher T.F., Kwon W.H. Minimum-energy asynchronous dissemination to mobile sinks in wireless sensor networks. Proceedings of the 1st International Conference on Embedded Networked Sensor Systems.

[19] Tunca C., Isik S., Donmez M.Y., Ersoy C. (2015). Ring Routing: An Energy-Efficient Routing Protocol for Wireless Sensor Networks with a Mobile Sink. IEEE Trans. Mob. Comput..

[20] Kim J.W., In J.S., Hur K., Kim J.W., Eom D.S. (2010). An intelligent agent-based routing structure for mobile sinks in WSNs. IEEE Trans. Consum. Electron..

[21] Lee J.H., Kim J.M., Jang B.T., Lee E.-S., Lee G., Howard D., Ślęzak D. (2011). Data Dissemination Protocol Based on Home Agent and Access Node for Mobile Sink in Mobile Wireless Sensor Networks. Proceedings of the 5th International Conference on Convergence and Hybrid Information Technology (ICHIT 2011).

[22] Jiang Y., Shi W., Wang X., Li H. (2014). A distributed routing for wireless sensor networks with mobile sink based on the greedy embedding. Ad Hoc Netw..

[23] Yang H., Ye F., Sikdar B. SIMPLE: Using Swarm Intelligence Methodology to Design Data Acquisition Protocol in Sensor Networks with Mobile Sinks. Proceedings of the 25th IEEE International Conference on Computer Communications.

[24] Yang W., Xing P., Liu Y. (2015). A positioning method of WSN based on self-adapted RSSI distance model. Chin. J. Sens. Actuators.

[25] Chen Y., Wang Z., Ren T., Lv H. (2015). Lifetime Optimization Algorithm with Mobile Sink Nodes for Wireless Sensor Networks Based on Location Information. Int. J. Distrib. Sens. Netw..

[26] Yang G., Xu H., He X., Wang G., Xiong N., Wu C. (2016). Tracking Mobile Sinks via Analysis of Movement Angle Changes in WSNs. Sensors.

[27] Ahmad A., Rathore M.M., Paul A., Chen B.W. (2015). Data Transmission Scheme Using Mobile Sink in Static Wireless Sensor Network. J. Sens..

[28] Konak A., Coit D.W., Smith A.E. (2006). Multi-objective optimization using genetic algorithms: A tutorial. Reliab. Eng. Syst. Saf..

[29] Maia G., Guidoni D.L., Viana A.C., Aquino A.L.L., Mini R.A.F., Loureiro A.A.F. (2013). A distributed data storage protocol for heterogeneous wireless sensor networks with mobile sinks. Ad Hoc Netw..

[30] Hu M., Wu T., Weir J.D. (2013). An Adaptive Particle Swarm Optimization With Multiple Adaptive Methods. IEEE Trans. Evol. Comput..

[31] Lin C.-J., Chen C.-H., Lin C.-T. (2009). A hybrid of cooperative particle swarm optimization and cultural algorithm for neural fuzzy networks and its prediction applications. IEEE Trans. Syst. Man Cybern. C Appl. Rev..

